# Living in each other’s pockets: insights into the life cycle of *Tremella caloplacae* s. l.

**DOI:** 10.3897/imafungus.17.157916

**Published:** 2026-02-13

**Authors:** Sandra Freire-Rallo, Veera Tuovinen Nogerius, Mats Wedin, Ana M. Millanes

**Affiliations:** 1 Instituto de Investigación en Cambio Global (IICG-URJC), Universidad Rey Juan Carlos, Tulipán s/n, 28933 Móstoles, Spain Departamento de Biología y Geología, Física y Química Inorgánica, Universidad Rey Juan Carlos (URJC) Tulipán Spain; 2 Departamento de Biología y Geología, Física y Química Inorgánica, Universidad Rey Juan Carlos (URJC), Tulipán s/n, 28933 Móstoles, Spain Instituto de Investigación en Cambio Global (IICG-URJC), Universidad Rey Juan Carlos Tulipán Spain; 3 Department of Ecology and Genetics, Uppsala University, Norbyvägen 18D 752 36 Uppsala, Sweden Uppsala University Uppsala Sweden; 4 Department of Botany, Swedish Museum of Natural History, PO Box 50007, SE-10405 Stockholm, Sweden Swedish Museum of Natural History Stockholm Sweden

**Keywords:** Basidiomycete, confocal laser scanning microscopy, fluorescent *in situ* hybridization, yeast

## Abstract

Complex life-cycles are common among fungi. Dimorphism in basidiomycetes involves alternation between a unicellular yeast phase and a filamentous phase, frequently forming basidiomata. Here we have studied the dimorphic life cycle of the lichen-inhabiting basidiomycetes in the *Tremella caloplacae* species complex, with particular focus on the newly distinguished *Tremella parietinae*. Using FISH-CLSM, PCR and Sanger sequencing, we have investigated the presence and distribution of the different life-cycle phases of *T. parietinae* within the lichen *Xanthoria parietina*, and also conducted an exploratory investigation into the presence of a *Tremella* yeast phase in other lichens of the *Teloschistaceae*. We could show that the filamentous phase of *T. parietinae* is restricted to the hymenium of *X. parietina*, whereas the yeast phase also grows elsewhere in the thallus. *Tremella caloplacae* s. str. is detected by PCR in *Calogaya*, *Flavoplaca* and *Gyalolechia* lichens, whereas its basidiomata are restricted to *Variospora* lichens. These findings suggests different lichen-specificity of *T. caloplacae* in the different phases of its life-cycle.

## Introduction

Fungal diversity is represented by a wide variety of morphologies, lifestyles, and nutritional habits (Hawksworth and Lücking 2017). Concerning nutritional habits, two big groups could be considered: saprotrophs and symbionts, which need to live in association with other living organisms. Among the latter, lichenized fungi live in symbiosis with algae or cyanobacteria, forming lichens. However, a great variety of bacteria and other fungi are known to live in association with lichens (Grimm et al. 2021). Lichen-associated fungi, ascomycetes or basidiomycetes (Diederich et al. 2025), spend their life cycle obligately in or on lichens, where they live as parasites, commensals or mutualistic symbionts. Within the currently accepted lichen-associated basidiomycetes, the most diverse group is the *Tremellomycetes*. *Tremellomycetes* are generally dimorphic, alternating between a unicellular yeast and a filamentous phase in their life cycle, presumably depending on the physical or chemical conditions of their environment (Bahn et al. 2005; Sia et al. 2000; Lin 2009; Hou et al. 2011). Within the *Tremellomycetes*, dimorphic species can be found in the orders *Cystofilobasidiales*, *Filobasidiales*, *Holtermanniales*, *Tremellales* and *Trichosporonales* (Bandoni 1995; Hibbett et al. 2007; Millanes et al. 2011; Liu et al. 2015), although the vast majority of species where dimorphism is known belong to the *Tremellales*. The tremellalean fungi have a great variety of nutritional habits, from saprotrophs to parasites of animals (including humans) or other fungi. Among the species associated with other fungi, the “jelly fungi” often form conspicuous and gelatinous basidiomata (sexual fruiting bodies), associated with corticioid fungi (e. g., *Tremella mesenterica*, associated with *Peniophora* spp.) (Bandoni 1987, 1995), whereas lichen-inhabiting species form basidiomata, which are comparatively small in size and often difficult to observe in the field (Diederich 1996; Diederich et al. 2022). Contrary to tremellalean animal parasites, where the yeast phase is usually better known, the filamentous stage has traditionally been more studied than the yeast stage in jelly fungi and in lichen-associated species. This filamentous phase is often visible as galls or deformations (containing the basidiomata) on the lichens (Diederich et al. 2022, 2025). In particular, numerous lichen-associated *Tremellomycetes* species were described in recent decades by studying the basidiomata that they form in the lichens with which they associate.

The presence of budding spores (i. e., a yeast stage) of basidiomycetes in lichens was already observed by Diederich (1996), but they were usually overlooked due to the limitations of conventional microscopy, and seldom isolated in culture (Prillinger et al. 1997; Cometto et al. 2022; Pérez et al. 2024). The studies by Spribille et al. (2016) and Tuovinen et al. (2019, 2021) were pioneering in imaging basidiomycete yeasts in lichens, using fluorescent *in situ* hybridization (FISH) coupled with confocal laser scanning microscopy (FISH-CLSM). Spribille et al. (2016) found *Cyphobasidium* (*Cystobasidiomycetes*, *Pucciniomycotina*) yeasts in a variety of macrolichen groups, especially in the family *Parmeliaceae*. In *Bryoria*, *Letharia*, *Alectoria*, *Hypogymnia* and *Usnea* species, the yeasts of *Cyphobasidium* were visualized in the cortex of specimens without the presence of galls. *Tremella lethariae* forms basidiomata on species of *Letharia*, but its yeast stage has also been detected in the cortex of several species of *Letharia* in specimens without basidiomatal galls (Tuovinen et al. 2019). Something similar occurs in *T. macrobasidiata* and *T. variae*, where the basidiomata are formed in the hymenium of *Lecanora chlarotera* and on the thallus of *L. varia*, respectively (Zamora and Perez-Ortega 2011; Zamora et al. 2016), but where the yeast stages have been visualized also across the areolae or the hymenia of specimens without basidiomata (Tuovinen et al. 2021). Tuovinen et al. (2021) suggested that the yeast phase could associate with a larger number of lichen species than the filamentous phase, that is, to be less specific. The same pattern occurs in the case of *Cyphobasidium hypogymniicola* and *Tremella hypogymniae*, where the presumable yeast phase has been reported from several macrolichen species, but so far, the filamentous phase is only known from *Hypogymnia physodes* (Millanes et al. 2016, 2025; Mark et al. 2020; Diederich et al. 2022).

**Figure 1.**
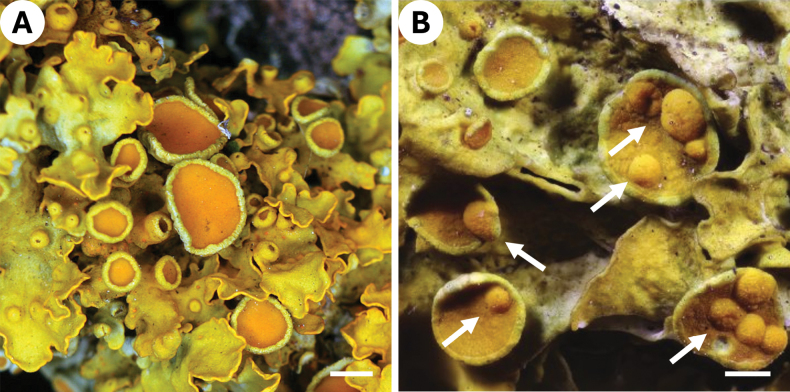
**A***Xanthoria parietina* hymenium without galls of *T. parietinae*. **B** Hymenium of *X. parietina* with galls (white arrows) of *T. parietinae* (Diederich et al. 2022; https://creativecommons.org/licenses/by-nc-sa/4.0/deed.en). Scale bars: 1 mm.

*Tremella* includes a great number of lichen-associated species with know­ledge-gaps about their life cycles, reproductive strategies, and species boundaries (Diederich 1996; Zamora et al. 2016; Tuovinen et al. 2019, 2021; Freire-Rallo et al. 2023a). *Tremella caloplacae* s. l. grows on lichens of the family *Teloschistaceae* and its filamentous phase is much better known than the yeast phase (Diederich 1996, 2007; Sérusiaux et al. 2003; Freire-Rallo et al. 2023b). This species was erroneously first described as a hyphomycete (Zahlbruckner 1906). Almost a century later it was recognized as a lichen-associated tremellomycete growing on several species of *Caloplaca* s. l. (Sérusiaux et al. 2003) and later reported on *Xanthoria* (currently *Rusavskia*) sorediata (Diederich 2007). Already Diederich (2007) suggested the possibility that *T. caloplacae* constituted a species complex, but it took almost two more decades to verify this (Freire-Rallo et al. 2023a, 2023b). Five new species have recently been formally described within the *T. caloplacae* species complex based on molecular data, morphology and lichen selection (Freire-Rallo et al. 2023b). *Tremella caloplacae* s. str. includes only specimens growing on *Variospora* spp. *Tremella elegantis* forms basidiomata in the hymenium of *Rusavskia elegans*, *T. nimisiana* in the hymenium of *Xanthocarpia* spp., *T. parietinae* in the hymenium of *Xanthoria parietina*, *T. pusillae* in the hymenium of *Calogaya pusilla*, and *T. sorediatae* on the thallus of *Rusavskia sorediata*. Although we can assume that they, like other *Tremellomycetes* and lichen-associated *Tremella* species, have a dimorphic life cycle (Diederich 1996; Chen 1998; Tuovinen et al. 2019, 2021), we want to investigate their life cycle further for several reasons: 1) with the exception of *T. sorediatae*, they are known to live exclusively in the hymenium of the lichens they associate with, 2) they live on *Teloschistaceae*, a lichen family producing anthraquinones – compounds in the thalline cortex with potential antifungal activity (Manojlovic et al. 2000; Scherrer et al. 2002; Brilhante et al. 2020; Raghuveer et al. 2023) - that could therefore limit the growth of the yeast phase in the thallus, and 3) *T. caloplacae* s. l. is not closely related to other lichen-inhabiting *Tremella* species that grow on *Parmeliaceae* or *Lecanoraceae*, and where the yeast phase has been thoroughly investigated. Among the species in the *T. caloplacae* complex, *T. parietinae* is particularly suited for such studies, since galls are only present in the hymenium of *Xanthoria parietina* (Fig. 1), which is a common and widespread lichen simplifying sampling in larger quantities.

In this work we will study the life cycle of *Tremella parietinae* and how it is co­nnected to *Xanthoria parietina*. A yeast stage in *T. parietinae* has never been clearly observed by optical microscopy so we will explore the location of the diffe­rent phases of the life cycle within the lichen thallus by FISH-CLSM, PCR amplification and Sanger sequencing. We will also investigate the specificity of representatives of the *T. caloplacae* complex towards the lichens with which they associate.

## Materials and methods

### Sampling for FISH

**Figure 2.**
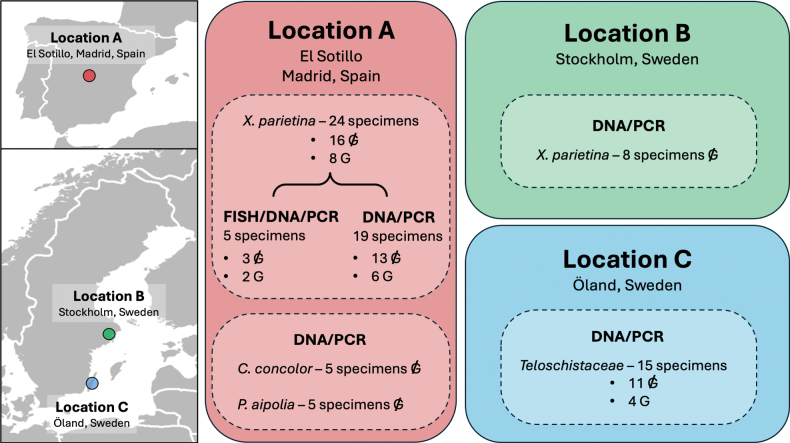
Sampling locations and specimens collected for analysis. The crossed-out G refers to collected specimens without galls, while G refers to collected specimens with galls.

To study the location of the different life-cycle phases of *Tremella parietinae* within *Xanthoria parietina*, we selected a population of *X. parietina* (location A, Spain, Suppl. material 3) with a high incidence of basidiomata-producing speci­mens. This population is situated in El Sotillo Recreational Area, an ecological corridor that forms a gradient of riparian vegetation from the Guadarrama River and its alluvial deposits with areas of *Arundo donax*, *Phragmites* spp., *Typha* spp., *Scirpus* spp., *Populus nigra*, *P. alba*, *Salix* spp., *Ulmus minor*, and *Fraxinus angustifolia* to forests of *Pinus pinea*, *P. pinaster* or *Quercus faginea*, and *Q. rotundifolia* typical of Mediterranean forests that can be found further away from the river. Specimens were collected in an easily accessible area (40°21'59.4"N, 3°56'43.1"W), dominated by *Fraxinus*, *Populus* and *Ulmus*, located in the vicinity of the M-501 motorway and on the outskirts of Villaviciosa de Odón (Madrid, Spain). We collected five fresh specimens of *X. parietina*, two with and three without basidiomatal galls (Fig. 2 and Suppl. material 3) from this population.

### Material fixation for FISH

The five specimens (Fig. 2 and Suppl. material 3) were collected after a period of rain and subsequently treated following the procedures in Tuovinen et al. (2021) to ensure that the lichens were metabolically active. Petri dishes were used to place them with moisturized filter paper, sprayed with _dd_H_2_O and loosely covered with a lid. Once the specimens dried up, they were moisturized again, replicating the process for seven days. Afterwards, they were moisturized one final time, and after that, we cut pieces of thalli by hand, apothecia without galls, and apothecia with galls from *Xanthoria parietina* specimens with galls. From *X. parietina* specimens without galls, we cut pieces of thalli and apothe­cia. Lichen pieces were placed in Eppendorf tubes with 1 ml of 1× PBS (pH 7.4) in a vacuum chamber. Vacuum was applied to the tubes with open lids for ca 10 s, then the tubes were closed and carefully tapped to help to remove the air from the fragments. Cycles of vacuum were applied until the lichen pieces sunk to the bottom of the tubes. PBS was replaced by 4% formaldehyde (methanol free) for fixation and incubated for 2 h at 4 °C. Fragments were then washed three times in 1× PBS for 10 min each, to remove traces of formaldehyde. Afterwards, fragments were incubated twice in acetone for 8 h to dissolve strongly autofluorescent (Suppl. material 1) secondary metabolites. The acetone was washed with 1× PBS as previously done, the PBS was removed, and the fragments were stored at −20 °C until FISH.

### Design of FISH probes

Specific probes for *Tremellales* (this study) and *Lecanoromycetes* (Spribille et al. 2016; Tuovinen et al. 2019) based on nuLSU sequences from *Tremella parietinae* and *X. parietina*, were designed and used following Tuovinen et al. (2021) (Table 1). Probes for *Tremellales* match multiple *Tremella* species from the *T. caloplacae* complex but not *Xanthoria parietina*; likewise, probes for the *Lecanoromycetes* match *X. parietina* while not *Tremella* species. *Tremella* species have strong autofluorescence in green wavelengths, whilst *X. parietina* and its photobiont are autofluorescent in red (hyphae and algae) and green (paraphyses, ascospores and algae) wavelengths (Suppl. material 1). To enhance the fluorescent signal intensity of the *Tremellales* probes, a double labelling with 6-FAM at 5’ end and FITC at 3’ end was performed. Each of the probes for the *Lecanoromycetes* were mono labelled with Cy5 at 5’ end.

**Table 1.** Probes and helper probes used for fluorescent *in situ* hybridization and confocal laser scan microscopy.

**Probe name**	**Probe sequence 5’–3’**	**Target/Comment**	**Source**
Tm_28SCalo	6-FAM-TATCACAGAGCGCCGGTC-FITC	* Tremellomycetes *	This study
Tm28SCalo_hlprA	GTCGTGTCAAGTACGGGA	Used with Tm_28SCalo	This study
Tm28SCalo_hlprB	CTCGACTCGTAGAAGA	Used with Tm_28SCalo	This study
Lec28S_1_Xan	Cy5-TCTGGTTGCAAGCGCTTC	* Lecanoromycetes *	Tuovinen et al. (2019)
Lec28S_1_Xan_hlprB	ACGGCTGATCACCCGCGG	Used with Lec28S_1_Xan	Tuovinen et al. (2019)
Lec28S_170_Xan	Cy5-CGAAGGGGTTTCACAC	* Lecanoromycetes *	Spribille et al. (2016)
Lec28SXan_170_hlprB	TGAGCTGCATTCCCAAAC	Used with Lec28S_170_Xan	Spribille et al. (2016)

### Fluorescent *in situ* hybridization

Fixed material of *Xanthoria parietina* was treated for permeabilization and hybridization as described by Tuovinen et al. (2019). To ensure that the probes entered the cells, the material was permeabilized for 1 h in 1× PBS at 36 °C with 0.1 mg/ml (0.04 U) chitinase from *Streptomyces griseus* and 1% SDS. Samples were then washed three times in 1× PBS for 10 min, subsequently dehydrated in 50%, 80% and 99.7% ethanol and let air-dry. For hybridization, the fragments were incubated for 3 h at 46 °C in a solution of 1 µM of each probe and helper probe, and hybridization buffer containing 10% formamide, 0.9 M NaCl, 0.01% SDS and 20 mM Tris-HCl (1 M pH 7.2). For each hybridization reaction, a negative control without probes was included. Mounting of the hybridized fragments was carried out with ProLong Glass Antifade (ThermoFisher) on microscopy slides.

### Confocal laser scanning microscopy and image processing

The CLSM of the hybridized slides was carried out by using an inverted IX83 Olympus confocal microscope with Olympus X-Line 60× NA 1.42 oil immersion objective. The autofluorescence of the specimens was tested with a Zeiss LSM710 confocal microscope, with Plan-Apochromat 63× NA 1.4 oil DIC M27 objective. Excitation of 6-FAM/FITC and Cy5 fluorophores was made with 405, 488 and 633 laser lines, respectively, with detection wavelengths of 493–598 nm for green and 638–797 nm for red. As both *Tremella parietinae* and *Xanthoria parietina* have strong autofluorescence in green and red, negative controls were used to set laser intensities and gain values to avoid interpretation of autofluorescence of the fragment as hybridization signal. Sequential scanning was used for Cy5 and 6-FAM/FITC. Z stacks of the fragments, with 1 mm thickness and 219 mm pinhole, were acquired with the microscope CellSens Dimensions software (Olympus).

Images were processed with Fiji in ImageJ (Schindelin et al. 2012; Schneider et al. 2012) or in ZenBlue (Zeiss). Sum slices projections and 3D projections were made, and color balance was adjusted for the clarity of presentation.

### Sampling for DNA extraction

Specimens for DNA extraction were collected in 3 different locations. Location A (El Sotillo, Madrid, Spain) that was previously described in the “Sampling for FISH” section. Location B (Stockholm, Sweden), is situated in an urban area in the vicinity of the Swedish Museum of Natural History and the metro station “Universitetet”. This location represents a population of *Xanthoria parietina* in which galls were not observed in any of the thalli. The vegetation is typical of urban areas, made up of *Acer platanoides*, *Betula pendula*, *Pinus sylvestris*, *Popu­lus tremula*, *Sorbus* spp., and *Tilia* spp. This area is heavily used by pedestrians and affected by traffic, the latter mainly coming from the nearby E18 motorway. Location C (Öland, Sweden) is composed by different localities in the island formed by calcareous cliff rocks in coastal environments. The island is formed by a bedrock of sedimentary calcareous limestones where vascular plants are scarce and the soil-crust is dominated by *Cladonia* spp., *Thamnolia vermicularis*, *Squamarina cartilaginea*, *Fulgensia* spp. and *Psora decipiens* (Albertson 1950; Fröberg 1999; Büdel et al. 2014).

To increase the sampling already achieved for FISH-CLSM, we collected a total of 24 fresh specimens (including the five specimens for FISH) from location A, eight with and 16 without galls; and eight specimens without galls from location B (Fig. 2 and Suppl. material 3). Since location A had a high incidence of basidiomata-producing specimens, we also checked for the presence of *Tremella parietinae* in other lichen species dominant in the locality, but not closely related to *Teloschistaceae* (i. e., *Candelariaceae* and *Physciaceae*). Five specimens of *Candelaria concolor* and five specimens of *Physcia aipolia* were collected for subsequent molecular analyses (Fig. 2 and Suppl. material 3), none of them with visible galls. In parallel, an exploratory screening for *Tremella caloplacae* s. l. was performed in related lichen species of the *Teloschistaceae* from which species in the *T. caloplacae* complex have been described. For this purpose, we collected 15 specimens of *Teloschistaceae* lichens from location C (Öland, Sweden) (Fig. 2 and Suppl. material 3). These were thoroughly examined for the presence of *T. caloplacae* s. l. Four of the specimens had *T. caloplacae* s. l. galls whilst 11 lacked them. No *Xanthoria parietina* specimens were collected from location C, since this lichen was not present in this locality.

### DNA extraction and amplification

**Figure 3.**
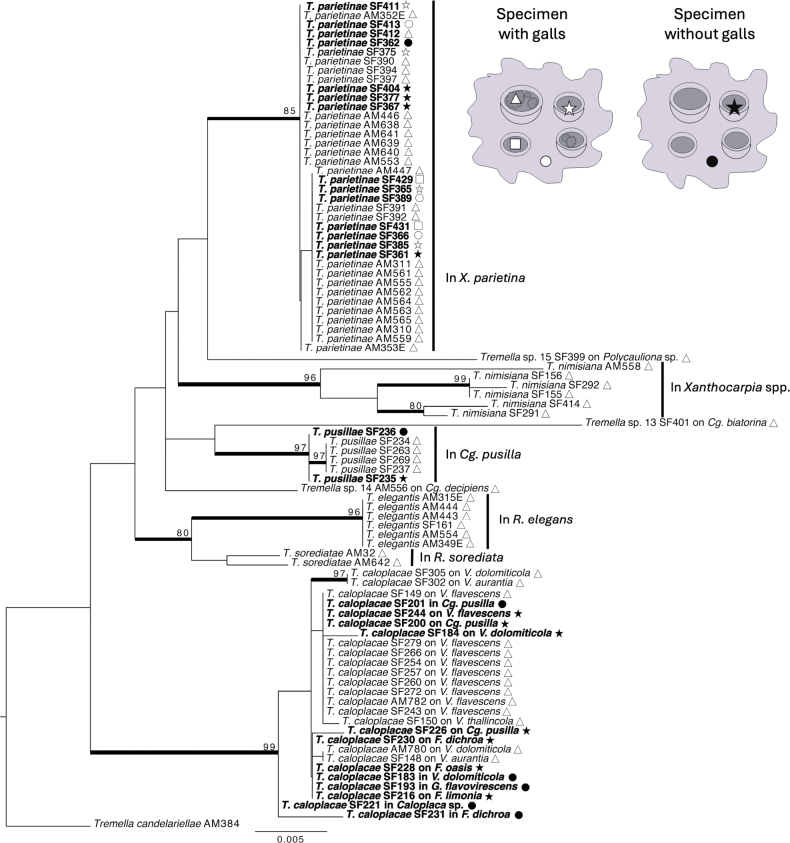
Phylogram based on ITS and nuLSU sequences, corresponding to the best tree recovered in the maximum likelihood analysis. A graphical legend with the symbols used in the terminals of the tree is included in the right upper corner of the figure. In all cases black symbols correspond to specimens without galls, and white symbols correspond to specimens with galls. Black circle: fragment from the thallus of a specimen without galls. Black star: complete apothecium (hymenium + thalline margin) of a specimen without galls. White circle: fragment from the thallus of a specimen with galls (galls in the hymenium). White star: complete apothecium without galls (hymenium + thalline margin) of a specimen with galls (galls in the hymenium). White square: hymenium without galls of a specimen with galls (galls in the hymenium). White triangle: galls (from the hymenium). Based on our FISH-CLSM results, we assume that sequences obtained from galls correspond to the filamentous phase, whereas sequences obtained from specimens or areas without galls, correspond to the yeast phase. ML-BS values ≥ 70 are indicated above boldface branches. Sequences newly produced in this study are indicated in bold font. Branch lengths are scaled to the expected number of substitutions per site.

DNA was extracted from the 57 specimens collected in populations A, B and C (Fig. 2 and Suppl. material 3), from thalli, hymenia, and complete apothecia (hymenium and thalline margin) of specimens without galls and from thalli, hymenia without galls, complete apothecia without galls (hymenium and thalline margin), and galls from specimens with galls (see legend in Fig. 3 and Suppl. material 3). PCR amplification and sequencing were used to 1) check the identity of the *Tremella* microscopic structures observed in *Xanthoria parietina* fragments by FISH-CLSM, since the FISH probes could potentially bind to any *Tremella caloplacae* s. l. species; 2) test the frequency of detection of *T. parietinae* in *X. parietina*; and 3) screen other lichens in the *Teloschistaceae* for the presence of lichen-associated *Tremella* species. For the *Candelaria concolor* and *Physcia aipolia* specimens collected from location A, thalli and complete apothecia were selected (Suppl. material 3). DNA was extracted by using the Qiagen DNeasy Plant MiniKit, following the manufacturer’s instructions but eluting twice with 50 µl of ultrapure water as final step.

PCR amplification of all DNA extractions was performed using specific primers for tremellalean fungi and *Tremella caloplacae* s. l., targeting the internal transcribed spacer I (ITS1), the 5.8S rDNA gene, the internal transcribed spacer II (ITS2) – from now on, the ITS region of ca. 500 nucleotides – and a portion of ca. 900 nucleotides of the nuLSU rDNA gene. PCRs were made by combining the specific primers for tremellalean fungi (BasidLSU3-3, BasidLSU1-5 (Millanes et al. 2011)), and specific primers for *T. caloplacae* s. l. (TRMcal_R2 and TRMLSU_1F (Freire-Rallo et al. 2023a)) with general fungal primers (ITS1F (Gardes and Bruns 1993) and LR5 (Vilgalys and Hester 1990)) (Suppl. material 4). Primer combinations and amplicon sizes were as follows: 1) ITS1F/BasidLSU3-3 amplifies ca. 900 nucleotides (complete ITS region and a fragment of ca. 400 nucleotides in the nuLSU region), 2) ITS1F/TRMcal_R2 amplifies ca. 800 nucleotides (complete ITS region and a fragment of ca. 300 nucleotides in the nuLSU region), 3) TRMLSU_1F/LR5 amplifies ca. 1200 nucleotides (ca. 300 nucleotides in the ITS region and a fragment of ca. 900 nucleotides in the nuLSU region), 4) BasidLSU1-5/LR5 amplifies ca. 1000 nucleotides of the nuLSU region, and 5) BasidLSU3-5/LR5 amplifies ca. 500 nucleotides in the nuLSU region.

PCRs were performed using Illustra^TM^ Hot Star PCR beads, according to the manufacturer’s instructions, with the following settings for each primer combination: 1) for ITS1F/BasidLSU3-3, ITS1F/TRMcal_R2, TRMLSU_1F/LR5 we run an initial denaturing at 95 °C for 5 min; 4 cycles of 95 °C for 40 s, 53 °C for 40 s and 72 °C for 90 s; 4 cycles of 95 °C for 30 s, 50 °C for 30 s and 72 °C for 90 s; 32 cycles of 95 °C for 30 s, 47 °C for 30 s and 72 °C for 90 s; final extension step of 72 °C for 10 min, and 2) for BasidLSU1-5/LR5 and BasidLSU3-5/LR5 we used an initial denaturing at 95 °C for 5 min; 4 cycles of 95 °C for 40 s, 56 °C for 40 s and 72 °C for 90 s; 4 cycles of 95 °C for 30 s, 53 °C for 30 s and 72 °C for 90 s; 32 cycles of 95 °C for 30 s, 50 °C for 30 s and 72 °C for 90 s; final extension step of 72 °C for 10 min. DNA amplification was performed by adding 5 µl of DNA extraction and 0.5 µl of each primer of a primer concentration of 10 µM, except for the gall fragments were we added 2 µl of DNA extraction. PCR amplification products were purified with Exo-sap-ITTM (USB Corporation, Cleveland, Ohio, USA) prior to sequencing. PCR products together with the primers for sequencing (Suppl. material 3) were sent for Sanger sequencing to Macrogen Spain (Barajas, Spain). These sequences, together with those from Freire-Rallo et al. (2023b) (Table 1), were used for the phylogenetic analysis.

### Sequence alignment and phylogenetic analysis

Assembling and editing of newly produced sequences was performed with Geneious Prime® 2021.0.3 (www.geneious.com). We generated a data matrix for the ITS and a portion of the nuLSU regions, combining the newly produced sequences with the sequences of *Tremella caloplacae* s. l. from Freire-Rallo et al. (2023b) (Suppl. material 5). For the phylogenetic analysis, *T. candelariellae* was used as an outgroup based on previous literature (Millanes et al. 2011; Liu et al. 2015; Freire-Rallo et al. 2023a). Sequences were aligned using the L-INS-I algorithm implemented in MAFFT (Katoh et al. 2002, 2005; Katoh and Standley 2013). We applied GBlocks v.0.91b (Castresana 2000) to identify and exclude misaligned positions, major insertions and ambiguous and/or divergent regions, with a relaxed selection of blocks as suggested by Talavera and Castresana (2007). We used Mesquite v.3.6 (Maddison and Maddison 2018) to check the alignments and convert terminal gaps to missing data. Maximum likelihood (ML) analyses were performed with IQTree (Nguyen et al. 2015) considering four independent partitions, ITS1, 5.8S, ITS2 and nuLSU. Conflicts among each individual partition were assessed with maximum likelihood ultrafast bootstrap in IQTree, considering highly supported clades (IQTree UF-BS > 95%) in disagreement an indication of conflict (Mason-Gamer and Kellogg 1996; Hoang et al. 2018). No conflict was detected in our data matrices; thus, we combined our four partitions in a single data set for subsequent analysis. ModelFinder in IQTree (Kalyaanamoorthy et al. 2017) was used to select the model for each partition, with the corrected Akaike information criterion (AICc): the TIMe + I model was selected for the ITS1, the K2P model for the 5.8S, the TIM2e + Γ4 for the ITS2, and the TIM3 + F + Γ4 for the nuLSU. Finally, a maximum likelihood search was performed, and support was achieved by standard bootstrap using 1000 bootstrap pseudoreplicates.

## Results

### FISH-CLSM studies in *Tremella parietinae*

**Figure 4.**
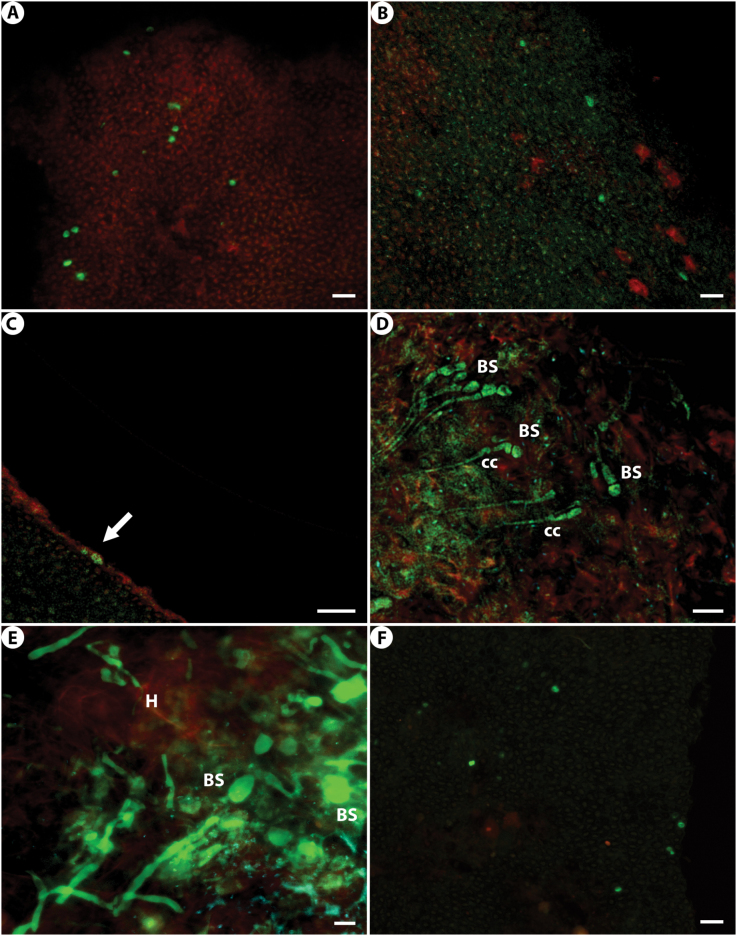
*Tremella parietinae* on *Xanthoria parietina* specimens, sum slides projection. Green: *T. parietinae*; red: algal autofluorescence and *X. parietina*. **A***Tremella parietinae* (SF403) yeasts in the lower cortex (thallus) of *X. parietina* (specimen without galls). **B***Tremella parietinae* (SF404) yeasts in the hymenium of *X. parietina* (specimen without galls). **C***Tremella parietinae* (SF408) yeasts in the thalline margin of an apothecium without galls from a specimen of *X. parietina* in which other apothecia had galls. Arrow indicates the position of yeasts. **D***Tremella parietinae* (SF411) filamentous stage in the hymenium of an apothecium without galls from a specimen of *X. parietina* in which other apothecia had galls. **E***Tremella parietinae* (SF409) filamentous stage in the hymenium of a *X. parietina* apothecium with galls. **F***Tremella parietinae* (SF413) yeasts in the upper cortex (thallus) of *X. parietina* (specimen with galls). BS: basidium. cc: clamp connection. H: Haustorium. Scale bars: 5 μm (**A–C, E, F**); 10 μm (**D**).

*Tremellales* probes designed to detect *Tremella parietinae* hybridize with the target species making it possible to discriminate between the fluorescent signal of the probes and the autofluorescence of *T. parietinae* itself. Cells of *Xanthoria parietina* and *T. caloplacae* s. l. were specifically stained in galls, apothecia without galls, and thalli from *X. parietina* specimens with and without galls. The pre­sence of *T. parietinae* was verified in the 12 studied fragments corresponding to thalli and apothecia from the 5 specimens of *X. parietina* with and without galls (Fig. 4, Suppl. material 2). Yeasts of *T. parietinae* were intermixed with hyphae of *X. parietina*, usually in superficial areas of both the upper and lower cortices of the thalli, the thalline margins of the apothecia, and the hymenium (Fig. 4, Suppl. material 2). The yeasts were distributed either grouped in small patches (Fig. 4A, Suppl. material 2) or in isolation (Fig. 4B, C, F, Suppl. material 2). The filamentous stage of *Tremella* was observed only in specimens with galls: in galls and in an apothecium without galls from one specimen of *X. parietina* with galls (Fig. 4D, E, Suppl. material 2). In these fragments we could observe hyphae, basidia, haustoria and clamp connections of *Tremella* (Fig. 4D, E), while basidiospores were only observed from *Tremella* galls (Suppl. material 2).

### DNA amplification and phylogenetic analysis

**Figure 5.**
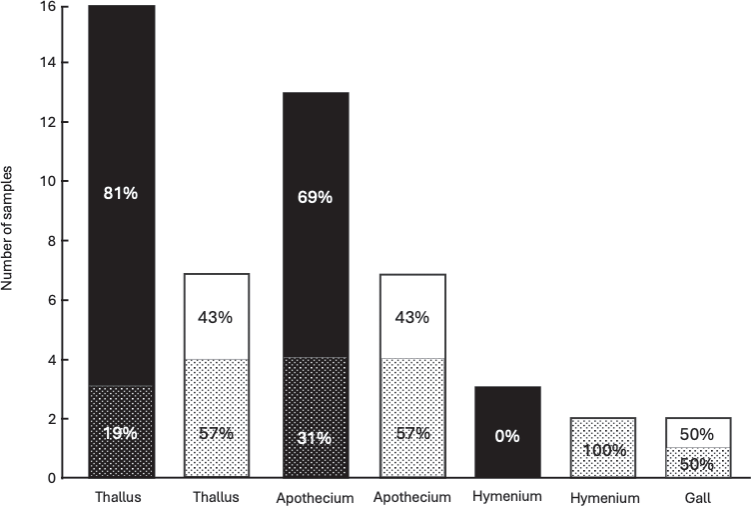
PCR detection (%) of *Tremella parietinae* in *Xanthoria parietina* fragments from specimens collected in location A. Black bars represent fragments from specimens without galls, and white bars represent fragments from specimens with galls. The dotted pattern represents the percentage of samples in which the detection was positive, while the solid color represents the percentage of negative detection. All thallus, apothecium and hymenium fragments were devoid of galls, regardless of whether the specimen they came from had galls.

We confirmed the identity of *Tremella parietinae* for all types of fragments studied with FISH except for those from the thallus of *Xanthoria parietina* without galls, from which no *Tremella* was amplified by PCR. From the 24 specimens of *X. parietina* collected in location A (Suppl. material 3), we detected *T. parietinae* in 5 of the 16 (ca. 38%) specimens without galls and in 7 of the 8 (ca. 88%) specimens with galls (Fig. 5). *T. parietinae* was not detected in any of the 8 specimens of *X. parietina* without galls collected from location B (Suppl. material 3). From the 15 specimens of *Teloschistaceae* lichens collected in location C (Suppl. material 3), we detected *T. caloplacae* s. str. (from *Athallia holocarpa*, *Calogaya arnoldii*, *Cg. pusilla*, *Caloplaca* sp., *Gyalolechia flavovirescens*, *Flavoplaca dichroa*, *F. limonia*, *F. oasis*, *Variospora dolomiticola*, *V. flavescens*), one *T. candelariellae* (from *X. parietina*), one *T. dendrographae* (from *X. parietina*), 16 *T. parietinae* (from *X. parietina*), and seven *T. pusillae* (from *Cg. pusilla*). In addition, five sequences corresponding to *T. candelariellae* were amplified from *C. concolor* (*Candelariaceae*) (Suppl. material 3). In the specimens of *Candelaria concolor* and *Physcia aipolia* collected in location A, no *T. parietinae* was detected in any of the fragments analysed, but *T. candelariellae* was amplified from all *C. concolor* specimens.

From the 41 sequences generated, we selected 32 sequences of good quality (18 of the ITS and 14 of the nuLSU regions) for the phylogenetic analyses. Ten sequences were extracted from *Teloschistaceae* specimens with galls and 22 from specimens without galls. Of these, 10 sequences corresponded to *Tremella caloplacae* s. str., 15 to *T. parietinae*, and seven to *T. pusillae*. Four of the 15 *T. parietinae* sequences obtained were from specimens also studied by FISH-CLSM (Figs 3, 4; Suppl. material 3): from a specimen of *Xanthoria parietina* without galls, one sequence from an apothecium; and from a specimen of *X. parietina* with galls, one sequence from an apothecium without galls, one sequence from a thallus and one sequence from a gall. *Tremella parietinae* was detected only in location A and exclusively in *X. parietina*, with or without galls. All newly generated sequences used for the phylogenetic analysis were deposited in GenBank (Suppl. material 3).

No incongruence was found among DNA regions and sequences were concatenated into a dataset for the ITS and nuLSU regions including the newly produced sequences (Suppl. material 3) in combination with ITS and nuLSU sequences from Freire-Rallo et al. (2023b). The data matrix was generated with a total of 1190 characters (ITS1: 1–119; 5.8S: 120–277; ITS2: 278–422; nuLSU: 423–1190). The best ML tree is shown in Fig. 3. Sequences of *Tremella* coming from specimens of *Xanthoria parietina* with or without galls group within the *T. parietinae* clade. Two sequences of *Tremella* obtained from specimens of *Calogaya pusilla* without galls (SF236 and SF235) are placed within the *T. pusillae* clade, whilst three other *Tremella* sequences coming from *Cg. pusilla* without galls grouped within the *T. caloplacae* s. str. clade (SF200, SF201 and SF226). The rest of the *Tremella* sequences extracted from different species of *Teloschistaceae* without galls all grouped within the *T. caloplacae* s. str. clade regardless of whether they come from thalli or apothecia fragments (Fig. 3).

## Discussion

### Location of the life-cycle phases of *Tremella parietinae*

*Tremella parietinae* is a dimorphic species, developing its filamentous phase exclusively in the hymenium of *Xanthoria parietina*. Our study confirms this in a population of *X. parietina*, where *Tremella* basidiomata are abundant (Fig. 4, Suppl. material 2, 3). The yeast phase, however, occurs both in the hymenium and the thallus (including thalline apothecium margins) of the lichens. That *T. parietinae* completes its life cycle within *X. parietina* was expected and is in line with the results obtained by Tuovinen et al. (2019, 2021) from other lichen-associated *Tremella* species. Although numerous studies have found *Tremellales* in environmental samples, there are no known records of *T. caloplacae* s. l. in such samples (Fernández-Mendoza et al. 2017; Núñez et al. 2017; Banchi et al. 2018; Li et al. 2020; Boekhout et al. 2022; Cometto et al. 2022; Mozzachiodi et al. 2022; Guerreiro et al. 2023; Pérez et al. 2024), which supports that the life cycle of *T. parietinae* is completed within *X. parietina*. Hyphae and a basidioma of *T. parietinae* have also been observed in a hymenium without galls from a specimen of *X. parietina* with galls (Fig. 4D). Similar to *T. parietinae*, other lichen-associated species of *Tremella* can develop basidiomata without visible galls like *T. candelariellae*, *T. endosporogena*, *T. protoparmeliae* or *T. rinodinae* (Diederich et al. 2022). Galls are never observed in *T. protoparmeliae*, despite the presence of mature intrahymenial basidiomata. Contrarily, *T. candelariellae*, *T. endosporogena*, and *T. rinodinae* initially develop intrahymenial basidiomata and the galls become visible only later (Diederich et al. 2022). In *T. parietinae*, the basidiomata observed in an apothecium without galls (Fig. 4D) have two-celled mature basidia that match the morphology of the basidiomata observed in galls (Freire-Rallo et al. 2023a), but from which no epibasidia (extension or final part of the basidium where meiosis takes place and spores are formed) have yet developed. Haustoria were not observed in this apothecium fragment, but clamp connections were present at the base of the mature basidia (Fig. 4D). Haustoria and basidiospores were only observed in basidiomata (Fig. 4E).

The presence of the yeast stage of *Tremella parietinae* in thalli (including thalline margins) and hymenia of lichens without galls has been verified by both FISH-CLSM and Sanger sequencing in five specimens from location A. The presence of yeasts in lichen thalli has also been confirmed by the same methods for other species like *T. macrobasidiata* and *T. variae*, in the thalli, thalline margin and hymenia of *Lecanora* (Tuovinen et al. 2021). These authors found that the unicellular *Tremella* stages were distributed throughout the thalli, from the upper to the lower cortices of the lichens. Contrarily, yeasts of *T. parietinae* are present in superficial or shallow parts of the upper and lower cortices but were not found within the algal layer or in the medulla. All this suggests, once again, that not only different lichen-inhabiting species have different ecological requirements, but also that the different phases of the life cycle of the same species thrive in different zones of the lichen thalli (Zamora and Perez-Ortega 2011; Zamora et al. 2016; Tuovinen et al. 2019, 2021).

It is well-known that basidiomycetes occur widely in lichens (Prillinger et al. 1997; Ekman 1999; Schneider et al. 2011; Spribille et al. 2016; Fernández-Mendoza et al. 2017; Banchi et al. 2018; Tuovinen et al. 2019, 2021; Tagirdzhanova et al. 2021, 2024; Cometto et al. 2022; Pérez et al. 2024; Kirkhus et al. 2025; Millanes et al. 2025). Our observations by FISH-CLSM suggest yeasts as the most plausible origin for the positive PCRs and sequences obtained, although the presence of hyphae or basidiomata cannot be fully discarded (Fig. 4).

The difference in *Tremella* DNA detection in populations A (El Sotillo, Spain) and B (Stockholm, Sweden) is remarkable. Presence of *T. parietinae* galls on *Xanthoria parietina* is very high in location A, while in location B, however, no speci­mens with galls on *X. parietina* were found. We detected *T. parietinae* in *X. parietina* specimens without galls in location A (Fig. 5), but no *Tremella* was discovered by PCR in any of the specimens studied from location B. *Hypogymnia* lichens from location B were also screened for yeasts of *Tremella hypogymniae* and *T. tubulosae* by Millanes et al. (2025), yielding very low detection rates. These observations suggest that some environmetal conditions at this location may constrain the presence or the detection of these yeasts. The lack of detection of *T. parietinae* and *T. caloplacae* s. l. cannot, however, be attributed to the actual absence with the current method, but could indicate false negatives obtained for different reasons (Burkardt 2000; Bacich et al. 2011; Tagirdzhanova et al. 2024, 2025). Indeed, we did not detect *Tremella* by PCR from all specimens where the presence was visible in the form of galls (Fig. 5). Bergmann and Werth (2017) had previously studied the presence of the lichenicolous species *Plectocarpon lichenum* and *T. lobariacearum* in *Lobaria* lichens by using Real-Time PCR (qPCR). They analyzed galls, fragments from adjacent areas to the galls and from areas without galls from lichen specimens with galls, as well as juvenile specimens without galls. They detected *T. lobariacearum* DNA in galls and fragments from areas adjacent to galls, but not from thalli lacking galls. Nevertheless, qPCR negative results were obtained for all types of fragments, including those of *T. lobariacearum* galls, indicative of technical issues leading to potential false negatives. Despite limitations of the PCR method, we detected *Tremella* yeasts in all type of lichen structures examined. This applies both to *T. parietinae* in *X. parietina* and to species of the *T. caloplacae* complex in *Teloschistaceae* lichens, indicating that the yeast stages of these species occur in the lichen thallus, apothecium and hymenium.

This is the first study focusing on *Tremella* species associated with lichens in the *Teloschistaceae*. These lichens are rich in the secondary compounds anthraquinones (Gaya et al. 2015; Llewellyn et al. 2023), which could have different influences on *Tremella* yeasts. On the one hand, anthraquinones are known to have antifungal activity (Basile et al. 2015; Raghuveer et al. 2023) and could have a defense-role against tremellalean or other potential fungal parasites. This hypothesis is supported by recent results showing a negative correlation between the presence of secondary metabolic compounds with antimicrobial effects, such as thiophaninic acid, fumarprotocetraric acid, or gyrophoric acid, and the presence of tremellalean fungi in some *Pertusaria* lichen species (Kirkhus et al. 2025). But anthraquinones also have a crucial role against ultraviolet (UV) radiation and blue light (Solhaug and Gauslaa 1996; Gauslaa and Margrete Ustvedt 2003). Since yeasts from other fungal groups are known to be sensitive to UV radiation (Alhamdy and Al-Sowayan 2020), the presence of anthraquinones could, in reality, protect the *Tremella* yeasts inside the *Xanthoria* thalli. These two hypotheses need further investigation.

The mechanisms behind the switching between life-cycle stages in lichen-associated species are not well understood. However, in other fungal species, such as those that are parasitic on cultivable plants and on animals, especially on humans, this change in morphology has been studied in greater depth (Boyce and Andrianopoulos 2015). Morphogenesis in these fungi has been related to different aspects such as propagation, infection, or evasion of host defense mechanisms (Nadal et al. 2008; Zhao et al. 2019). Some factors that can induce morphological change in dimorphic pathogenic fungi are known, such as temperature increase for thermodimorphic fungi, pH fluctuations, changes in nutrient availability, presence of compounds generated by the host immune defenses or recognition between compatible yeasts through pheromones (Nadal et al. 2008; Cervantes-Chávez et al. 2009; Martínez-Soto and Ruiz-Herrera 2013; Boyce and Andrianopoulos 2015; Gauthier 2015; Zhao et al. 2019). Nevertheless, for lichen-associated dimorphic fungi, the optimal conditions for the development of their sexual reproductive structures, such as the formation of basidiomata, remain unknown. In location A, there is a high abundance of *X. parietina* and these have an unusually high presence of *Tremella* galls. The causes behind this high incidence of galls in this population are not known, but it would not be strange to think that a high incidence of yeasts within the lichen thalli would provide opportunities for mating of compatible yeast strains, maximizing the chance of chemical and physical interaction among them. To our knowledge, there are no studies on the physiological or developmental mechanisms behind the induction or formation of galls. However, studies on *Plectocarpon* species suggest that the presence of these structures is related to changes in the production of secondary metabolites in the lichen (Merinero et al. 2015; Asplund et al. 2016). Asplund et al. (2018) suggest that the presence of *P. lichenum* reduces the synthesis of carbon-based secondary compounds (CBSCs) and the growth of *Lobaria pulmonaria*, but it is still to be clarified if this CBSCs reduction is caused by the presence of *P. lichenum*. The high incidence of *T. parietinae* yeasts in location A combined with a hypothetical optimal combination of environmental factors could favor the formation of the filamentous phase, and thus, the high incidence of observed galls.

### Specificity towards the lichen

Despite the high incidence of *Tremella parietinae* in location A, the species was not detected by PCR or sequencing in other lichen species present and abundant in the locality (i. e., *Candelaria concolor* and *Physcia aipolia*). In the exploratory study carried out by screening other *Teloschistaceae*, the presence of *T. caloplacae* s. l. was detected by PCR in fragments of thalli and apothecia without galls from specimens with and without galls (Suppl. material 3). Moreover, *Tremella* sequences obtained from specimens of *Calogaya*, *Flavoplaca* and *Gyalolechia* are placed within *T. caloplacae* s. str., where the formation of basidiomata is restricted to species of *Variospora* (Freire-Rallo et al. 2023b). That *T. caloplacae* s. str. is present in other *Teloschistaceae* species besides *Variospora* spp., could indicate a lower specificity towards the lichen in the yeast phase, although we cannot discard that these detections could correspond to early stages of the filamentous phase. Basidiomycete yeast phases are seen as widespread and less specific than their filamentous phases (Fernández-Mendoza et al. 2017; Tuovinen et al. 2019, 2021; Mark et al. 2020; Kirkhus et al. 2025). In our investigations in *C. pusilla*, *Variospora* spp. and *Xanthoria parietina* lichens without galls, other lichen-associated *Tremella* species − the basidiomata of which is not known from these lichens − namely *T. dendrographae*, *T. candelariellae* or *T. caloplacae* s. str. have also been detected (Suppl. material 3). That *T. caloplacae* s. l. and *T. candelariellae* are phylogenetically closely related but the lichens they associate with are phylogenetically distant, could be attributed to events of speciation driven by ‘host’ switching as is the case for the species within the *T. caloplacae* complex (Freire-Rallo et al. 2023b). Within the same lichen specimen, more than a single tremellomycete species could be present (Tuovinen et al. 2019, 2021). *Tremella lethariae. T. macrobasidiata* and *T. variae* are such examples (Tuovinen et al. 2019, 2021), where the yeast phase has been found in species other than the lichen in which they develop their filamentous phase (Diederich 1996; Zamora and Perez-Ortega 2011; Millanes et al. 2016; Zamora et al. 2016). Nevertheless, the detection of more than one lichen-associated species is limited when performing PCR analyses, and the presence of several species could be misinterpreted as contaminations (Ekman 1999; Černajová and Škaloud 2019). The DNA proportion of a biomass-wise dominating *Tremella* could mask the presence of other lichen-associated *Tremella* species, thus resulting in a lower detection rate (Tuovinen et al. 2021). Still, Tuovinen et al. (2019) sequenced *T. lethariae* and a different lineage of *Tremella* informally called *Tremella* sp. B (Lindgren et al. 2015) from the same *Letharia* specimens. In recent years, metagenomic studies have shown a great diversity of basidiomycete yeasts in lichens (Lendemer et al. 2019; Smith et al. 2020; Tagirdzhanova et al. 2021, 2024; He et al. 2022), due to the sensitivity and the decrease in price of these techniques. Metagenomic analyses could likewise improve our understanding of lichen selection of the yeast phase of *Tremella* species.

## Conclusions

This is the first study investigating the life cycle and the specificity of *Tremella* species associated with lichens of the *Teloschistaceae*, and detecting *Tremella* yeasts in these lichens, when galls are not present. We have confirmed that *Tremella parietinae* is a dimorphic species, developing its filamentous phase exclusively in the hymenium of *Xanthoria parietina*. The yeast phase is present in both the hymenium and the thallus suggesting lower specificity of the yeast regarding its location within the lichen environment. In addition, *T. caloplacae* s. str. is present in several *Teloschistaceae* species whereas the basidiomata are only known from *Variospora* spp. Although we cannot assume with certainty that these DNA detections correspond exclusively to the *Tremella* yeast phase, our overall findings support the growing evidence on the lower specificity of the yeast phases of lichen-associated tremellalean fungi compared to their filamentous phases. All in all, our study paves the way for more comprehensive studies into the life cycle and ecological roles of these fungi.

## Additional information

### Use of AI

No use of AI was reported.

### Adherence to national and international regulations

All the fungal strains used in this study have been legally obtained, respecting the Convention on Biological Diversity (Rio Convention).

### Author ORCIDs

Sandra Freire-Rallo https://orcid.org/0000-0003-1576-0507

Veera Tuovinen Nogerius https://orcid.org/0000-0002-8558-3289

Mats Wedin https://orcid.org/0000-0002-8295-5198

Ana M. Millanes https://orcid.org/0000-0001-5003-4186

### Data availability

All of the data that support the findings of this study are available in the main text or Supplementary Information.

## Supplementary materials

Supplementary material 1 Autofluorescence of *Xanthoria parietina* Data type pdf https://binary.pensoft.net/file/1540264This dataset is made available under the Open Database License (http://opendatacommons.org/licenses/odbl/1.0/). The Open Database License (ODbL) is a license agreement intended to allow users to freely share, modify, and use this Dataset while maintaining this same freedom for others, provided that the original source and author(s) are credited. Sandra Freire-Rallo, Veera Tuovinen Nogerius, Mats Wedin, Ana M. Millanes

Supplementary material 2 *Tremella parietinae* on *Xanthoria parietina*, sum slides projection Data type pdf https://binary.pensoft.net/file/1540265This dataset is made available under the Open Database License (http://opendatacommons.org/licenses/odbl/1.0/). The Open Database License (ODbL) is a license agreement intended to allow users to freely share, modify, and use this Dataset while maintaining this same freedom for others, provided that the original source and author(s) are credited. Sandra Freire-Rallo, Veera Tuovinen Nogerius, Mats Wedin, Ana M. Millanes

Supplementary material 3 PCR detection of *Tremella* in the studied lichen specimens and selected fragments Data type xlsx https://binary.pensoft.net/file/1540266This dataset is made available under the Open Database License (http://opendatacommons.org/licenses/odbl/1.0/). The Open Database License (ODbL) is a license agreement intended to allow users to freely share, modify, and use this Dataset while maintaining this same freedom for others, provided that the original source and author(s) are credited. Sandra Freire-Rallo, Veera Tuovinen Nogerius, Mats Wedin, Ana M. Millanes

Supplementary material 4 Primers used for the DNA amplification of ITS and LSU regions Data type xlsx https://binary.pensoft.net/file/1540267This dataset is made available under the Open Database License (http://opendatacommons.org/licenses/odbl/1.0/). The Open Database License (ODbL) is a license agreement intended to allow users to freely share, modify, and use this Dataset while maintaining this same freedom for others, provided that the original source and author(s) are credited. Sandra Freire-Rallo, Veera Tuovinen Nogerius, Mats Wedin, Ana M. Millanes

Supplementary material 5 Specimen information Data type xlsx https://binary.pensoft.net/file/1540268This dataset is made available under the Open Database License (http://opendatacommons.org/licenses/odbl/1.0/). The Open Database License (ODbL) is a license agreement intended to allow users to freely share, modify, and use this Dataset while maintaining this same freedom for others, provided that the original source and author(s) are credited. Sandra Freire-Rallo, Veera Tuovinen Nogerius, Mats Wedin, Ana M. Millanes
