# Supplementary material for: Living in each other’s pockets: insights into the life cycle of Tremella caloplacae s. l
Source: IMA Fungus. 2026 Feb 13;17:e157916. doi: 10.3897/imafungus.17.157916 (PMC12924053; doi:10.3897/imafungus.17.157916)
Supplement: Supplementary material 1 — Autofluorescence of Xanthoria parietina [file imafungus-17-e157916-s001.pdf]

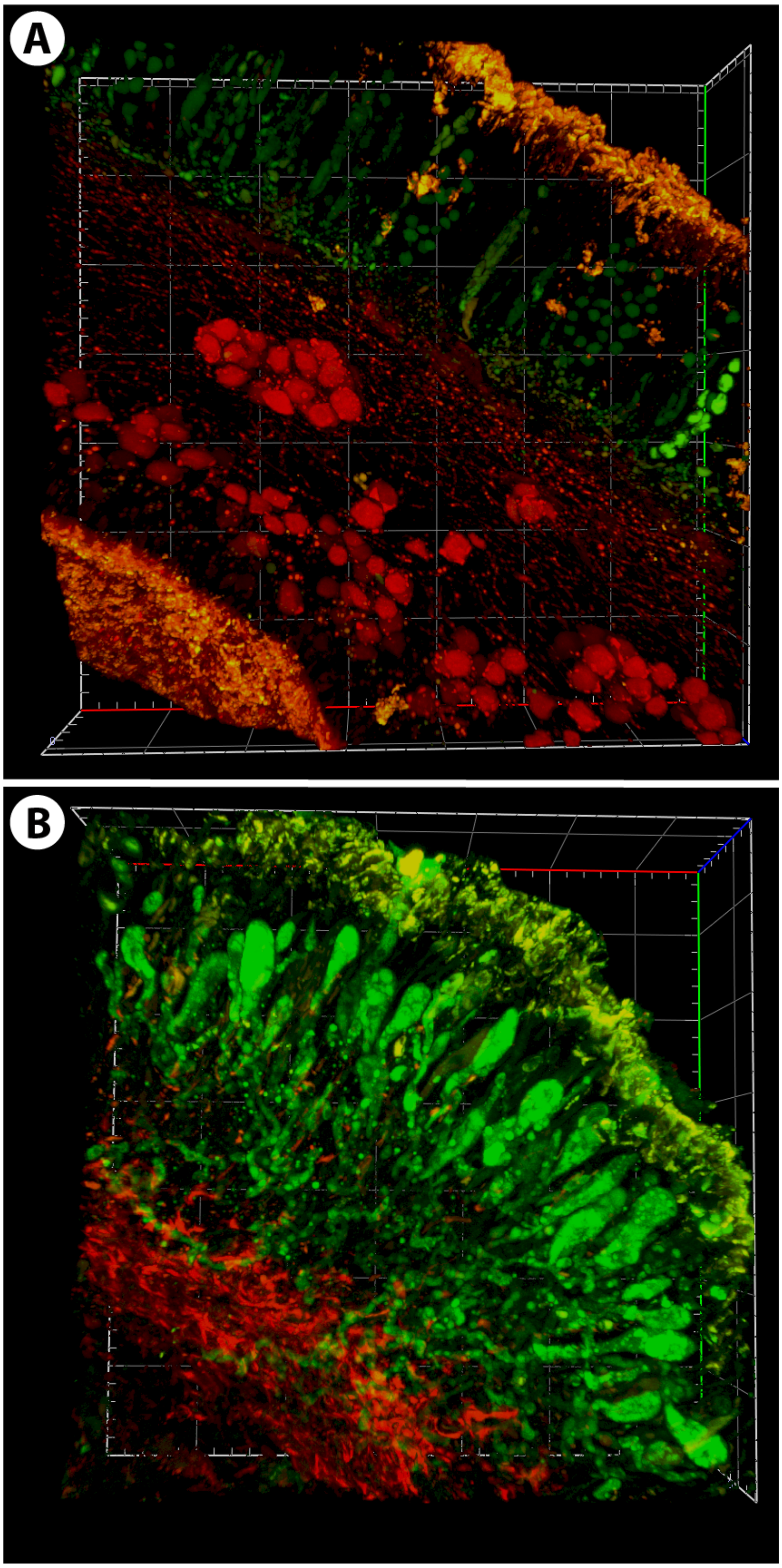
A
B

Figure S1. Autofluorescence of *Xanthoria parietina* (A) and *Tremella parietinae* (B) before acetone treatment. A) Green: autofluorescence of *X. parietina* ascospores; red: autofluorescence of algae and *X. parietina* hyphae; orange-yellow: autofluorescence of parietin crystals from green and red wavelengths, becoming yellow in overlay. B) Green: autofluorescence of *T. parietinae*; red: autofluorescence of algae and *X. parietina* hyphae; green-yellow: autofluorescence of parietin crystals from green and red wavelengths becoming yellow in overlay.
