# Supplementary material for: Living in each other’s pockets: insights into the life cycle of Tremella caloplacae s. l
Source: IMA Fungus. 2026 Feb 13;17:e157916. doi: 10.3897/imafungus.17.157916 (PMC12924053; doi:10.3897/imafungus.17.157916)
Supplement: Supplementary material 2 — Tremella parietinae on Xanthoria parietina, sum slides projection [file imafungus-17-e157916-s002.pdf]

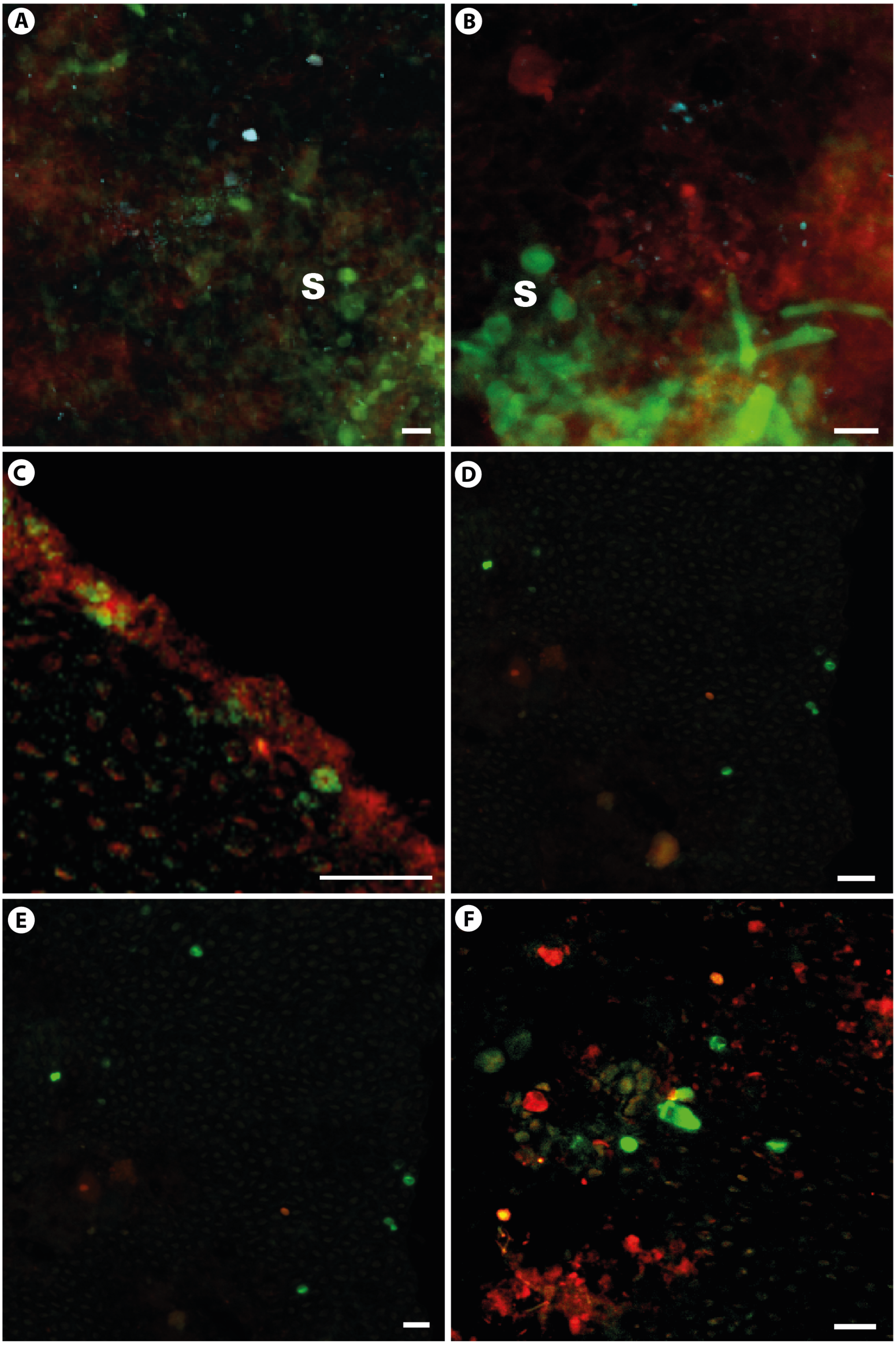

Figure S2. *Tremella parietinae* (A-B: SF412; C: SF408; D: SF404; E-F: SF405) on *Xanthoria parietina*, sum slides projection. Green: *T. parietinae*; red: algal autofluorescence and

X. parietina. A, B) Filamentous stage of *T. parietinae* in the hymenium of a *X. parietina* specimen with galls. C) Yeast of *T. parietinae* in the upper part of the thalline margin of a *X. parietina* specimen with galls. D) Yeast of *T. parietinae* in the hymenium of a *X. parietina* specimen without galls. E) Yeast of *T. parietinae* in the upper cortex of a *X. parietina* thallus, specimen without galls. F) Yeast of *T. parietinae* in the lower cortex of a thallus, *X. parietina* specimen without galls. S: basidiospores. Scale bars: 5 µm.
